# Bilateral thalamic infarction following cerebral venous thrombosis in a patient with ulcerative colitis

**DOI:** 10.1002/ccr3.5404

**Published:** 2022-02-15

**Authors:** Jayant Kumar Yadav, Gaurav Nepal, Aakar Thapa, Sandip Jaiswal, Shreejana Thapa, Avinash Chandra

**Affiliations:** ^1^ Department of Neurology Annapurna Neurological Institute and Allied Sciences Kathmandu Nepal; ^2^ Department of Internal Medicine Maharajgunj Medical Campus Tribhuvan University Institute of Medicine Kathmandu Nepal; ^3^ Department of Internal Medicine Patan Academy of Health Sciences Lagankhel Nepal

**Keywords:** cerebral venous sinus thrombosis, cerebral venous thrombosis, thalamic infarct, ulcerative colitis

## Abstract

Cerebral Venous Thrombosis is a rare extra‐intestinal manifestation of ulcerative colitis. Ulcerative colitis is a hypercoagulable state and, if poorly managed, can predispose to thrombosis, including thrombosis of the cerebral veins.

## Graphical abstract

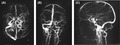

## 1 INTRODUCTION

Venous Thrombosis (CVT) is an important cause of stroke in young adults caused by complete or partial occlusion of the major cerebral venous sinuses or the smaller feeding cortical veins. The estimated prevalence of CVT is 1.3–1.6 cases per 100,000 people and accounts for 0.5% of all stroke cases.^1^ Compared with the general population, the incidence of CVT is higher in children and women. CVT can be caused by different conditions such as infectious, structural, hypercoagulable states, hematological, hormonal, collagen, vascular diseases, and oral contraceptive pills among other causes.^2^ However, in rare cases, inflammatory bowel diseases (IBD), such as Crohn's disease and ulcerative colitis (UC), have also been reported to predispose CVT.^3^ We herein present an unusual case of a young woman with UC under treatment presenting with CVT and bilateral thalamic infarction.

## 2 CASE PRESENTATION

A 30‐year‐old female patient referred from a suburban hospital of western Nepal presented to the emergency department in July 2021, with the chief complaint of left‐sided weakness, which she noticed on waking up. She mentioned that before waking up with weakness, she had experienced a gradual onset debilitating frontal headache for the preceding 4 days not relieved with over‐the‐counter medications (Paracetamol and ibuprofen). The headache was continuous, of throbbing nature but not associated with fever, visual or sensory aura, photophobia, nausea, or vomiting. There was no diurnal or nocturnal variation in headache, not associated with neck rigidity; however, it was associated with bilateral ocular pain without double vision or vision loss. Her weakness was progressive, gradually involving the right side of the body, and was associated with an inability to speak and bladder/bowel incontinence. There was no history of ear /nasal discharge, dental infection, cough, sinus pain, surgery, or trauma. Further elaboration of history revealed that she had been diagnosed with UC 18 months back and was on immunosuppression under prednisolone and azathioprine. She had no abdominal complaints, including diarrhea recently. She had no other comorbidities and was doing fine until this event. She is a non‐smoker, non‐alcoholic. She was using a levonorgestrel implant as a birth control method for the last 3 years. Her menstrual period was regular. She had a positive family history of stroke in her mother in her 50s.

At presentation, her score on the National Institute of Health Score Scale (NIHSS) was 7 and on the modified Rankin Scale (mRS) was 2. Her vital signs were within normal limits. During the examination, she was disoriented. Power was reduced in bilateral lower limbs (4^+^/5) whereas it was normal in bilateral upper limbs (5/5). The sensation was not assessable, neck rigidity was absent, and reflexes were intact. The plantar response was flexor bilaterally. Thorough cranial nerves and eye examination were unremarkable, and there was no noticeable change in fundoscopy. She had no cognitive deficits. Systemic examinations were within normal limits.

**FIGURE 1:**
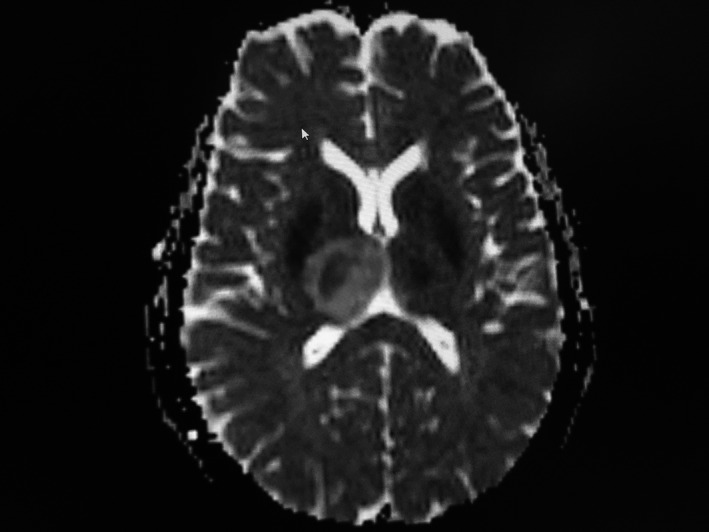
ADC map brain showing bilateral thalamic infarcts, right thalamus is more involved left

**FIGURE 2:**
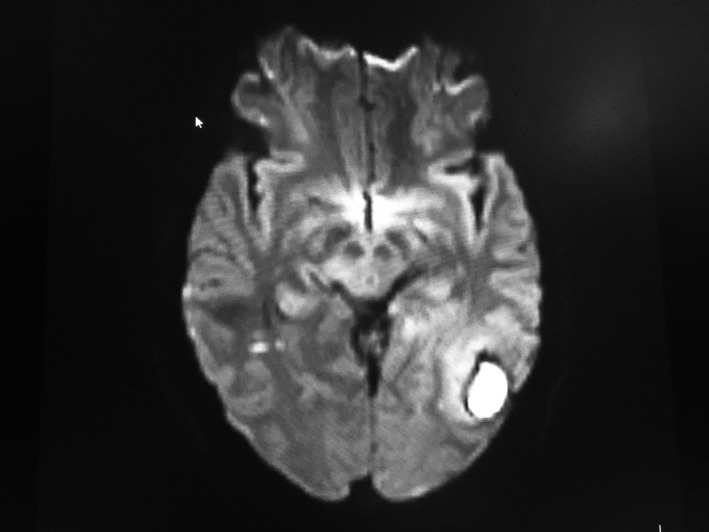
MRI brain showing intracerebral hematoma in the left parieto‐occipital region

**FIGURE 3:**
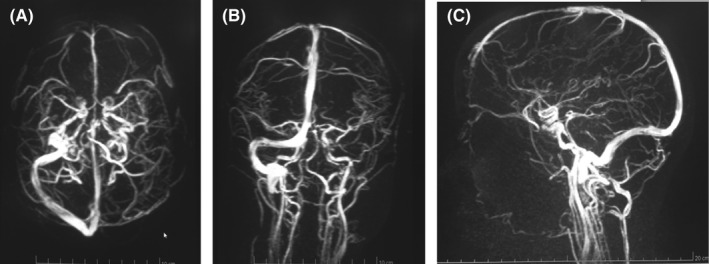
MRV showing thrombosis of sigmoid sinus (A), left transverse sinus (B), and straight sinus (C)

The hemogram, baseline coagulation profile were normal; however, D‐dimer (2.16 µg/ml), Erythrocyte Sedimentation rate (ESR) (50 mm/h), and C‐reactive protein (CRP) (25 mg/dl) were raised. The coagulation parameters including factor level, homocysteine, and antithrombin III levels could not be measured due to financial constraints. Her cardiac enzymes, renal function test, liver function test, lipid profile, and thyroid function tests were within normal limits. The nasopharyngeal swab Polymerase Chain reaction (PCR) for the Severe Acute Respiratory Syndrome Coronavirus‐2 (SARS CoV‐2) virus was negative. With the suspicion of CVT, plain and contrast‐enhanced Magnetic Resonance Imaging (MRI) of the brain with Magnetic Resonance Venography (MRV) was sent. Infarction involving the bilateral thalamus (Right > Left) was seen in Attenuated Diffusion Coefficient (ADC) map (Figure 1) and 2.3 × 2.6 × 1.5 cm hematoma in the left parieto‐occipital region with thin extension to the adjacent area of the left temporal lobe and perilesional edema with some mass effect (Figure 2). Thrombosis of the dural venous sinus thrombosis involving sigmoid dural venous sinus (Figure 3A), left transverse sinus (Figure 3B), and the straight sinus (Figure 3C) was evident.

She was admitted to the neurology ward with the diagnosis of bilateral thalamic infarction with dural venous thrombosis due to a hypercoagulable state secondary to UC. She was treated with subcutaneous Heparin 40 U and monitored with activated partial thromboplastin time (APTT), intravenous levetiracetam, intravenous dexamethasone, intravenous mannitol, and intravenous pantoprazole. Regular Azathioprine and steroids were continued for UC. Once her condition stabilized, regular physiotherapy and acupuncture therapy were done. Her hospital stay was uneventful. At discharge, the patient was stable with an NIHSS score of 2 and a mRS score of 1; a motor power of 5/5 in the right upper/lower limbs and left upper limb, whereas it was 4/5 in the left lower limb. She was sent home on oral dabigatran and oral levetiracetam. All her regular medications were continued at this time. A week following discharge, she developed nausea, vomiting, and multiple episodes of diarrhea and was advised to visit her gastroenterologist. Colonoscopic examination with biopsy revealed severely active pancolitis. She underwent laparoscopic total proctocolectomy with ileal pouch‐anal anastomosis with diverting ileostomy. She was recovering well during follow‐up.

## 3 DISCUSSION

Ulcerative colitis is a type of inflammatory bowel disease that causes inflammation and ulcers in the colon and rectum. It is associated with several extra‐intestinal manifestations including arthritis, uveitis, erythema nodosum, pyoderma gangrenosum, primary sclerosing cholangitis, among several other conditions.^4^ Patients with inflammatory bowel disease are at an increased risk for both venous and arterial thromboembolism. This risk appears to be increased in patients with pan‐colonic disease and those with ulcerative colitis. Hypercoagulable states associated with UC are often serious, recurrent, tend to occur at unusual sites (e.g., cerebral vein), are associated with active disease, and occur in younger individuals. CVT is a rare but severe complication of UC. Most commonly, the superior sagittal and lateral sinuses are involved.^2^ Furthermore, it has been associated with the use of steroids, possibly indicating an active underlying disease. Our patient was a young woman (in her 30s), who had ulcerative colitis flare despite steroids and azathioprine therapy.

What increases the chances of hypercoagulation in UC is not known. Data suggests that it is multifactorial and includes elevated levels of factors V, VIII, fibrinogen, total homocysteine, and decreased levels of antithrombin III and platelet disorders. Thrombocytosis and leukocytosis are common.^5^ Assessment of coagulation factors, homocysteine, and antithrombin III could not be done in our patient due to unaffordability and was unlikely to alter our treatment plan.

She was started on early anticoagulation with heparin. The European guidelines recommend treating acute CVT with heparin at a therapeutic dosage even in patients with intracerebral hemorrhage at baseline followed by oral anticoagulants for 3–12 months.^6^ With bridging anticoagulation, our patient recovered fully or her symptom was improved. She was commenced on oral dabigatran on discharge. This is the standard practice unless there is a contraindication to anticoagulation such as active bleeding. Her risk factors for coagulopathy included her positive family history of stroke in her mother and her diagnosis of UC. Her flare of ulcerative colitis increased the risk of thromboembolism. For patients with acute severe UC who fail to respond to steroids and immunosuppressant, surgery is indicated.^7^ Total proctocolectomy often cures the patient of ulcerative colitis. However, a case has been reported in a patient who developed CVT 10 years after total proctocolectomy.^8^ This case also highlights the importance of regular follow‐up in such a patient to monitor the disease activity. Our patient had missed several appointments due to COVID pandemic.

Furthermore, thalamic infarction following CVT is rare and is sparsely reported in the literature. It may be either arterial or venous in origin. Occlusion of the artery of Percheron is responsible for arterial ischemia, whereas venous infarction may be caused by thrombosis of the straight sinus, where the posterior group of thalamic veins drains. This is due to upstream venous pressure. Our patient had thrombosis of the straight sinus and was responsible for bilateral thalamic infarction. It is important to differentiate between these two, as arterial occlusion requires immediate thrombolysis, whereas venous occlusion requires anticoagulation.^9^

## 4 CONCLUSION

Cerebral sinus vein thrombosis is a rare but often fatal complication of ulcerative colitis if undiagnosed. Cerebral venous sinus thrombosis may rarely cause bilateral thalamic infarction. Patients with ulcerative colitis should follow up with their regular physician to track activity of the disease.

## CONFLICT OF INTEREST

None of the authors has any conflict of interest to disclose.

## AUTHOR CONTRIBUTIONS

JKY, GN, SKJ, ST, AK, and AC were involved in drafting the manuscript. All except GN was involved in the management of the case. All authors approved the final draft for publication.

## ETHICAL APPROVAL

Ethical approval of case report is not needed in accordance with local ethical guidelines.

## CONSENT

The patient provided written informed consent for publication of this case report and accompanying images.

## Data Availability

Not applicable.

## References

[1] Ferro JM , Bousser MG , Canhão P , et al. European stroke organization guideline for the diagnosis and treatment of cerebral venous thrombosis – endorsed by the European Academy of Neurology. Eur J Neurol. 2017;24(10):1203‐1213. 28833980 10.1111/ene.13381

[2] Saposnik G , Baarinagarrementeria F , Brown RD , et al. American Heart Association Stroke Council and the Council on Epidemiology and Prevention. Diagnosis and management of cerebral venous thrombosis: a statement for healthcare professionals from the American Heart Association/American Stroke Association. Stroke. 2011;42(4):1158‐1192. doi:10.1161/STR.0b013e31820a8364 21293023

[3] Kupfer SS , Rubin DT . Inflammatory bowel disease and cerebral venous sinus thrombosis. Gastroenterol Hepatol (N Y). 2006;2(12):914‐917. 28331482 PMC5359939

[4] Vavricka SR , Schoepfer A , Scharl M , Lakatos PL , Navarini A , Rogler G . Extraintestinal manifestations of inflammatory bowel disease. Inflamm Bowel Dis. 2015;21(8):1982‐1992. 26154136 10.1097/MIB.0000000000000392 PMC4511685

[5] Irving PM , Pasi KJ , Rampton DS . Thrombosis and inflammatory bowel disease. Clin Gastroenterol Hepatol. 2005;3(7):617‐628. 16206491 10.1016/s1542-3565(05)00154-0

[6] Ferro JM , Bousser MG , Canhão P , et al. European Stroke Organization guideline for the diagnosis and treatment of cerebral venous thrombosis – Endorsed by the European Academy of Neurology. Eur Stroke J. 2017;2(3):195‐221. 31008314 10.1177/2396987317719364 PMC6454824

[7] Andersson P , Söderholm JD . Surgery in ulcerative colitis: indication and timing. Dig Dis. 2009;27(3):335‐340. doi:10.1159/000228570 19786761

[8] Yerby MS , Bailey GM . Superior sagittal sinus thrombosis 10 years after surgery for ulcerative colitis. Stroke. 1980;11(3):294‐296. 7394868 10.1161/01.str.11.3.294

[9] Hoitsma E , Wilmink JT , Lodder J . Bilateral thalamic infarction may result from venous rather than arterial obstruction. J Stroke Cerebrovasc Dis. 2002;11(1):47‐50. 17903855 10.1053/jscd.2002.123975

